# Physical Fitness and Mitochondrial Respiratory Capacity in Horse Skeletal Muscle

**DOI:** 10.1371/journal.pone.0034890

**Published:** 2012-04-18

**Authors:** Dominique-Marie Votion, Erich Gnaiger, Hélène Lemieux, Ange Mouithys-Mickalad, Didier Serteyn

**Affiliations:** 1 Equine European Centre of Mont-le-Soie, University of Liege, Vielsalm, Belgium; 2 Equine Clinic, Faculty of Veterinary Medicine, University of Liege, Liege, Belgium; 3 Department of Visceral, Transplant, and Thoracic Surgery, D. Swarovski Research Laboratory, Medical University of Innsbruck, Innsbruck, Austria; 4 Centre of Oxygen, Research and Development, University of Liege, Liege, Belgium; University of Texas Health Science Center at San Antonio, United States of America

## Abstract

**Background:**

Within the animal kingdom, horses are among the most powerful aerobic athletic mammals. Determination of muscle respiratory capacity and control improves our knowledge of mitochondrial physiology in horses and high aerobic performance in general.

**Methodology/Principal Findings:**

We applied high-resolution respirometry and multiple substrate-uncoupler-inhibitor titration protocols to study mitochondrial physiology in small (1.0–2.5 mg) permeabilized muscle fibres sampled from *triceps brachii* of healthy horses.

Oxidative phosphorylation (OXPHOS) capacity (pmol O_2_•s^−1^•mg^−1^ wet weight) with combined Complex I and II (CI+II) substrate supply (malate+glutamate+succinate) increased from 77±18 in overweight horses to 103±18, 122±15, and 129±12 in untrained, trained and competitive horses (*N* = 3, 8, 16, and 5, respectively). Similar to human muscle mitochondria, equine OXPHOS capacity was limited by the phosphorylation system to 0.85±0.10 (*N* = 32) of electron transfer capacity, independent of fitness level. In 15 trained horses, OXPHOS capacity increased from 119±12 to 134±37 when pyruvate was included in the CI+II substrate cocktail. Relative to this maximum OXPHOS capacity, Complex I (CI)-linked OXPHOS capacities were only 50% with glutamate+malate, 64% with pyruvate+malate, and 68% with pyruvate+malate+glutamate, and ∼78% with CII-linked succinate+rotenone. OXPHOS capacity with glutamate+malate increased with fitness relative to CI+II-supported ETS capacity from a flux control ratio of 0.38 to 0.40, 0.41 and 0.46 in overweight to competitive horses, whereas the CII/CI+II substrate control ratio remained constant at 0.70. Therefore, the apparent deficit of the CI- over CII-linked pathway capacity was reduced with physical fitness.

**Conclusions/Significance:**

The scope of mitochondrial density-dependent OXPHOS capacity and the density-independent (qualitative) increase of CI-linked respiratory capacity with increased fitness open up new perspectives of integrative and comparative mitochondrial respiratory physiology.

## Introduction

Muscle contraction requires ATP, and oxidative phosphorylation (OXPHOS) is the dominant pathway for providing energy during aerobic exercise and for recovery after anaerobic exercise. The level and contribution of OXPHOS to muscle energy supply depends on the type, intensity and duration of exercise (for a review, see [1]). These functional differences are well established by comparison of aerobic *versus* anaerobic athletic species [2], [3] or sports athletes [4], [5], and glycolytic (white) *versus* aerobic (red) muscles and muscle fibres [6], [7]. In contrast, limited information is available on the functional characteristics of OXPHOS and differences in mitochondrial respiratory control in skeletal muscle of various species and at different levels of training [8]. Comparative physiology aims at elucidating the functional and integrative mechanisms of complex systems such as mitochondria. In addition, evaluation of altered mitochondrial function in health and disease may provide the key to improve our knowledge of pathophysiology as a basis to develop novel therapies.

Recent investigations on mitochondrial respiratory control revealed fundamental differences of mitochondrial physiology in skeletal muscle of humans *versus* rodents (reviewed by [8]). These surprising differences of mitochondrial respiratory control have been masked by conventional protocols of bioenergetics, using Complex I (CI) or Complex II (CII) linked substrates separately for measurement of active and passive respiration (ADP-stimulated and ADP-limited; States 3 and 4, respectively [9]). Under these conditions, substrate supply to selected segments of the electron transfer system (ETS) exerts artificial control over respiration which prevents physiological control mechanisms from being expressed. Maximum OXPHOS capacities are obtained in mitochondrial preparations only on the basis of physiological substrate combinations linked to electron transfer through CI and CII simultaneously (CI+II), required to reconstitute tricarboxylic acid (TCA) cycle function [8], [10]. Convergent electron input through CI+II into the Q-junction exerts an additive effect on respiration with the consequence of a shift of metabolic control from external substrate supply to catalytic capacities distributed throughout the OXPHOS system [8], [10]–[13].

Maximum OXPHOS capacity in muscle with a physiological substrate cocktail has not yet been studied on a larger comparative scale of different mammalian species [14]. Equine skeletal muscle is of primary interest in comparative physiology for the following reasons: besides the pronghorn antelope and dogs, horses are among the most athletic species [15], [16]. These active animals consume more oxygen per unit body mass than any other mammal. Because horses are large, the oxygen consumed *in toto* at maximal exercise intensity is several-fold greater reaching more than 100 liters per minute (for a review, see [17]). Indeed, maximal oxygen consumption per body mass (specific 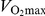) is more than twice as high in race horses compared to elite human athletes (>200 ml.min^−1^.kg^−1^ [18] *versus* 80 ml.min^−1^.kg^−1^ [19]). The up to 50-fold increase in 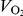 in rest-work transitions is achieved as a result of remarkable cardiopulmonary adaptations: horses reach their outstanding 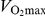 mainly by achieving superlative rates of oxygen delivery [17]. This exceptional aerobic capacity of horses has been gained over time by evolutionary selection of these herbivores whose survival was dependent directly on speed to escape predators and on endurance to find food and water. After domestication, selection for high aerobic capacity has been continued by specific breeding programs. Depending on their breed and training, the contemporary horse may excel in different types of disciplines requiring endurance and/or speed aptitudes. Endurance horses may race 160 km at a mean speed of 20 km/h and thoroughbred race horses run at high speed (∼60 km/h) over distances up to 5 km.

In horses, the major sources of energy for muscle contraction are intramuscular glycogen and triglycerides, as well as blood-borne glucose and fatty acids. The predominant sources of energy and relative contribution of aerobic and anaerobic metabolism depend on exercise type and training (for a review in horses, see [7]).

In equestrian disciplines requiring stamina but also in thoroughbred racing requiring speed aptitude, aerobic phosphorylation contributes predominantly to ATP replenishment during exercise. Indeed, thoroughbreds race from 1000 to 3000 m, distances that cannot be run on the sole resort of anaerobic pathways [20].

In horses, muscle oxidative capacity has been classically evaluated by determining the activity of mitochondrial marker enzymes such as succinate dehydrogenase or mitochondrial volume density [21]–[24]. These histological methods do not reveal information about integrative mitochondrial function. The first direct measurement of respiratory capacity with a Clark oxygen sensor was performed on mitochondria isolated from equine muscle samples collected before and after exercise with the percutaneous needle biopsy method [25]. This study showed that exercise depresses transitorily muscle respiratory capacity with NADH-linked (CI) substrates. Application of conventional mitochondrial respiratory protocols with substrate supply linked either to CI or CII and restriction to States 3 and 4 have largely masked the potential diversity of mitochondrial respiratory control patterns in skeletal muscle of different species and at different levels of fitness. Determination of muscle respiratory capacity with physiological substrate cocktails is warranted to improve our knowledge of mitochondrial physiology in horses.

Recently, a preliminary study demonstrated the feasibility to monitor mitochondrial respiration in small samples of permeabilized muscle fibres with high-resolution respirometry (HRR) in horses [26]. Determination of OXPHOS capacity and respiratory control patterns in horse skeletal muscle for further comparative studies requires the development of protocols that are identical, as far as possible, in applications to different species, but which are also rigorously tested in and adapted to the specific constraints and peculiarities of this large animal. The present investigation on horse skeletal muscle has tested the methodology used to study mitochondrial function in muscle microbiopsies by HRR [27] to define reference protocols for horses (from sampling procedure to data analysis). In addition, this study applied these protocols to a large group of horses to address the following questions: (1) What is the upper limit of tissue-OXPHOS capacity in *triceps brachii* muscle of healthy horses and how does it scale with body size and outstanding aerobic capacity of horses? (2) Is maximum OXPHOS capacity influenced by training status and does it contribute to performance? (3) To which extent are mitochondrial respiratory control patterns in horse skeletal muscle comparable to skeletal muscle mitochondria of mouse, rat and man?

## Materials and Methods

### Animals and muscle microbiopsy sampling procedure

All procedures of this study were approved by the Animal Ethic Commission of the University of Liege (agreement n°07–629). Muscle microbiopsies were obtained from 32 healthy horses (Table 1). Horses were not owned by the University of Liege but belonged to individual owners who gave their consent for the horses to be biopsied. The body condition score (BCS) according to Arnaud *et al*. (1997) [28] results from an evaluation of the mass of fat deposits in five specific body locations by palpation, complemented by visual assessment of seven anatomic sites. Each evaluation receives a notation and the average defines the BCS ranging from 0 (emaciated) to 5 (obese). Four fitness groups were studied, ranging from untrained overweight, normal BCS untrained, trained and competitive horses (Table 1).

**Table 1:**
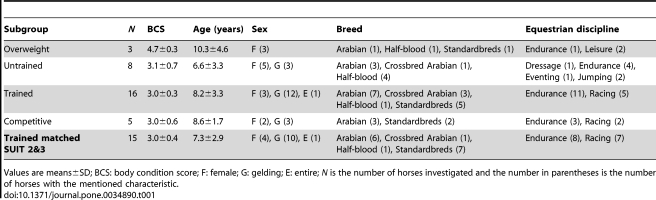
Characteristics of horses used in the study.

Values are means±SD; BCS: body condition score; F: female; G: gelding; E: entire; *N* is the number of horses investigated and the number in parentheses is the number of horses with the mentioned characteristic.

Studies on muscle energetics in horses are primarily based upon the use of percutaneous needle biopsies [21], [29], [30] which provide 50 to 150 mg of muscle wet weight (*W*_w_) for biochemical, histochemical, immunohistochemical, and morphological studies. Although this quantity appears to be small compared to hundreds of kg of muscle of horses, the technique is quite invasive and is therefore difficult to apply as a routine procedure, especially for sport horses. In search for a minimally-invasive procedure, we applied the microbiopsy technique [31] to collect on average 20 mg from the *triceps brachii* muscle using a 14 G biopsy needle mounted on an automatic instrument (Pro-Mag™ Ultra Biopsy Instrument, Angiotech, Gainesville, Fl, USA). Fibre-type distribution is not homogenous within the muscle [32], [33] thus requiring an accurate standardization of the sampling site and depth for consistency of interpretation of results [34]. Briefly, the sampling site was shaved (one cm^2^), the skin was desensitized by subcutaneous injection of 0.5 ml of mepivacain (scandicaine 2%, AstraZeneca, Brussels, Belgium), and aseptically prepared. Mepivacaine has been used in our study because it is commonly used by veterinary practitioners and it is thus readily available. Local anaesthetics have to be injected strictly under the skin since they may induce alteration of muscle mitochondrial energetics [35], [36]. Muscle microbiopsy specimens were taken at 50 mm depth in the long head of the *triceps brachii* through a skin incision made with the tip of a scalpel blade nr 11, such that a direct effect of the mepivacain injection on the mitochondria of the muscle biopsy can be practically excluded. Closure of the skin incision was not necessary, the microbiopsy procedure was completed within 15 minutes, was well tolerated by horses and sedation was not required. Two to three muscle samples of, on average, 19.9±5.1 mg *W*_w_ each were obtained via the same skin opening and transferred immediately into ice-cold relaxing solution BIOPS [37], [38] containing 10 mM CaK_2_-EGTA, 7.23 mM K_2_-EGTA, 20 mM imidazole, 20 mM taurine, 50 mM K-MES, 0.5 mM dithiothreitol, 6.56 mM MgCl_2_, 5.77 mM ATP and 15 mM phosphocreatine adjusted to pH 7.1. The fibres were kept at 4°C until further preparation.

### Permeabilized muscle fibre preparation

After transferring the tissue sample into ice-cold BIOPS, connective tissue was removed and the muscle fibres were mechanically separated using two pairs of forceps with sharp tips. Complete permeabilization of the plasma membrane was ensured by gentle agitation for 30 min at 4°C in 2 ml of BIOPS solution containing 50 µg/ml saponin. The fibre bundles were rinsed by agitation for 10 min in ice-cold mitochondrial respiration medium (MiR05; 0.5 mM EGTA, 3 mM MgCl_2_, 60 mM K-lactobionate, 20 mM taurine, 10 mM KH_2_PO_4_, 20 mM Hepes, 110 mM sucrose and 1 g/l BSA essentially fatty acid free adjusted to pH 7.1 [39]. The permeabilized muscle fibres (Pfi) were immediately used for HRR.

### High-resolution respirometry

One to 2.5 mg *W*_w_ (microbalance; METTLER TOLEDO, Zaventem, Belgium) of Pfi were added to each Oxygraph-2k chamber (OROBOROS INSTRUMENTS, Innsbruck, Austria) containing 2 ml of MiR05 at 37.0°C maintained constant at ±0.001°C by electronic Peltier regulation. Oxygen concentration, *c*_O2_ (µM), and oxygen flux per muscle mass (pmol O_2_•s^−1^•mg^−1^ *W*_w_) were recorded online using DatLab software (OROBOROS INSTRUMENTS, Innsbruck, Austria).

After calibration of the oxygen sensors at air saturation, a few ml of oxygen gas was introduced into the gas phase above the stirred aqueous phase in the partially closed chambers to reach a concentration of 500 µM O_2_. During the complete substrate-uncoupler-inhibitor titration (SUIT) protocols (about 1 hour), the oxygen level in the chambers was maintained between 200 and 500 µM O_2_ to avoid any oxygen limitation of respiration [27]. Whereas air-level oxygen pressure is not a limiting factor for respirometric studies with isolated mitochondria, low oxygen levels (<200 µM O_2_) have to be avoided with Pfi because of restricted oxygen diffusion [40]. Such oxygen dependence was also observed in the present study with horses (*data not shown*) and thus, the oxygraph chambers were reoxygenated when the O_2_ level in the medium dropped towards 200 µM. Respiratory flux was corrected on-line for instrumental background, determined at experimental oxygen levels [41].

Three SUIT protocols were applied to obtain extended information on coupling and substrate control parameters [27]. From the 32 horses included in the study, 15 were used for simultaneous application of all three SUIT protocols.

**Figure 1:**
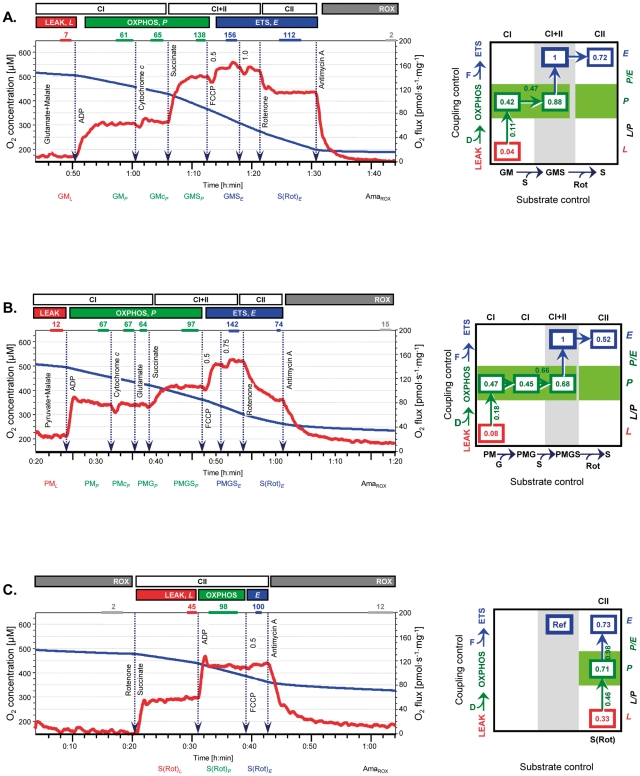
Respirometric protocols with permeabilized fibres from horse skeletal muscle (*triceps brachii*). **A**: SUIT1 protocol with electron flow through CI supported by glutamate+malate. **B**: SUIT2 protocol with electron flow through CI supported by pyruvate+malate. **C**: SUIT3 protocol with electron flow through CII. ***Left panels:*** Oxygen concentration (µM; blue lines) and muscle mass-specific oxygen flux (pmol O_2_·s^−1^·mg^−1^ wet weight; red lines). Marked sections correspond to steady-state fluxes at different coupling states (*L*, *P* and *E*) and substrate states. Numbers on top of traces are ROX-corrected tissue mass-specific fluxes at various metabolic states, and ROX (pmol O_2_·s^−1^·mg^−1^). ***Right panels:*** Coupling/substrate control diagrams with flux control ratios (*FCR*) normalized relative to ETS capacity with convergent CI+II electron input (values framed). Values linked to vertical and horizontal arrows are coupling control ratios (*L/P* and *P/E* ratio), and substrate control ratios (CI/CI+II in the OXPHOS state). *FCR* linked by arrows directly to the reference state are also coupling control ratios (*P/E*) or substrate control ratios (CII/CI+II). Whereas internal *FCR* are obtained in SUIT 1 and 2 (A and B), the *FCR* for SUIT3 (C) are calculated for the externally determined reference state (Ref in C averaged from SUIT1 and 2 protocols).

In SUIT1 (Figure 1A), electron flow through CI was supported by the NADH-linked substrates glutamate+malate (GM; 10 and 2 mM) with subsequent addition of ADP (2.5 mM). ADP-stimulated respiration with various substrate combinations (State 3 [9]) represents OXPHOS capacity (State *P*), if limitation by ADP or oxygen is strictly avoided [8]. High ADP concentrations are required to overcome the low affinity for ADP in Pfi as compared to isolated mitochondria [42]. In SUIT2, the initial substrates were pyruvate+malate (PM; 5 and 2 mM) with subsequent stimulation of OXPHOS by glutamate (10 mM; G; Figure 1B). Under aerobic conditions, pyruvate is converted to acetyl-CoA by the pyruvate dehydrogenase (PDH) complex with concurrent reduction of NAD^+^ [43]. The PDH complex of eukaryotic cells is located in the mitochondrial matrix and thus, is not lost during permeabilization. Surprisingly, addition of 5 mM pyruvate to glutamate+malate exerted a significant inhibition of OXPHOS capacity, but this effect was not seen upon stepwise titration of pyruvate (*data not shown*), indicating the importance of optimizing tissue-specific details of SUIT protocols before initiating an experimental series.

The next steps were identical in both protocols, adding succinate (S; 10 mM) for convergent electron flow through CI+II into the Q-junction (supported by GMS or PMGS in SUIT1 or 2, respectively). The capacity of the phosphorylation system (adenine nucleotide translocase, inorganic phosphate transporter, and ATP synthase) may limit OXPHOS capacity with an apparent excess capacity of the ETS over the phosphorylation system [8], [10]–[12]. This was tested by stepwise addition of the uncoupler FCCP (0.05 µM, followed by 0.025 µM steps until maximal oxygen flux was reached), obtaining ETS capacity, *E*, with convergent electron flow through CI+II. Higher concentrations of FCCP were inhibitory. For clarification, therefore, the noncoupled state of ETS capacity is distinguished from generally uncoupled respiratory states with various degrees of activation or inhibition [27]. Electron input into the Q-junction through CII alone was subsequently induced by inhibition of CI by rotenone (Rot; 0.5 µM). Finally, residual oxygen consumption (ROX) was obtained by addition of antimycin A (2.5 µM) to block electron transfer through Complex III (CIII). Inhibition requires a long time to be reached with the sole addition of this inhibitor in human [12] and horse fibres (this is tissue- and species specific). In our study, we provided the necessary time to obtain steady-state ROX. Oxygen fluxes were corrected by subtracting ROX from each measured mitochondrial steady-state. Antimycin A is not the only choice to obtain ROX after inhibition of the ETS at the level of CIII. Myxothiazol (0.5 µM; not used in the present study) provides an alternative [27]. Inhibition of Complex IV (CIV) by cyanide or azide cannot be used in our protocols because cyanide inhibition is reversed by keto acids pyruvate and α-ketoglutarate, added in our experiments and produced in the TCA cycle, respectively. In addition, the reversibility of this inhibition is particularly effective at the high oxygen levels required in experiments with permeabilized fibres [27], [44].

Integrity of the outer mitochondrial membrane was tested by adding 10 µM cytochrome *c* [45] after ADP in the presence of substrates feeding electrons into CI. The cytochrome *c* test was applied on all samples in SUIT1 and 2. Injury of the outer mitochondrial membrane leads to loss of cytochrome *c* from the mitochondria, and to significant stimulation of respiration following addition of exogenous cytochrome *c* to the respiration medium.

An additional protocol (SUIT3; Figure 1C) was applied with the single substrate succinate (10 mM) in the presence of rotenone (0.5 µM), obtaining a sequence of coupling states for CII-respiration in the absence of adenylates (LEAK), stimulated by 2.5 mM ADP (OXPHOS), and uncoupled by 0.05 µM FCCP (ETS; additional FCCP titrations did not increase flux). Finally, ROX was obtained with 2.5 µM antimycin A.

The average 20 mg *W*_w_ of muscle tissue collected per microbiopsy was sufficient to perform three SUIT protocols using 1.0 to 2.5 mg of Pfi per chamber. This amount was small compared to the (at least) 100 mg of muscle needed to isolate mitochondria for functional studies [46]. About 1.5 mg of Pfi were found to be the ideal amount of muscle tissue to be used for respirometric studies with healthy horses, to obtain a good resolution of flux and yet avoiding a too rapid decline of oxygen concentration which would require frequent reoxygenations.

Oxygen flux was expressed as tissue mass-specific respiration (per mg *W*_w_), or as flux control ratios, *FCR*, with internal normalization for maximum ETS capacity in each experimental run. Depending on the protocol, the *FCR* (ranging from 0 to 1) are a function of coupling control only (SUIT3), or are a complex function of coupling and substrate control. Coupling control ratios, *CCR*, such as the LEAK control ratio, *L/E*, and phosphorylation system control ratio, *P/E*, are obtained at constant substrate supply, whereas substrate control ratios, *SCR*, are flux ratios at constant coupling state [8].

In a preliminary experiment, three untrained horses were sampled to test the preservation of muscle samples over time (SUIT1 and 2). Five microbiopsy specimens of muscle per horse were taken at 50 mm depth via the same skin opening. Each specimen was transferred immediately into 10 ml BIOPS solution and kept at 4°C until preparation for respirometry measurements that were performed from day 1 (as soon as possible after sampling) to day 5 in a random order. Respirometry measurements of each specimen were performed in duplicate for each protocol.

### Statistical analyses

All results are presented as mean±SD. The Wilcoxon signed-rank and Mann-Whitney-Wilcoxon tests were used to compare paired data (*i.e.* respirometric parameters among the same horses: OXPHOS and ETS capacities obtained with CI+II substrate combinations in SUIT1 and 2 and S(Rot) in coupled and/or noncoupled states in SUIT1, 2 and 3) and unpaired data (*i.e.* horses with different fitness level: optimal BCS and untrained *versus* optimal BCS and trained *versus* competitive horses with optimal BCS in SUIT1: OXPHOS and ETS capacities, *FCR* and *SCR*), respectively. A value of *P*<0.05 was considered as level of significance. Statistical analyses to test preservation of muscle tissue over time were performed using SAS (SAS 9.1, procedure GLM). After checking for normality, a linear model with the fixed effects of day was used for each respirometric parameter, *SCR* and *FCR*.

## Results

### Effects of training and body condition score on mitochondrial respiratory capacity and control

**Figure 2:**
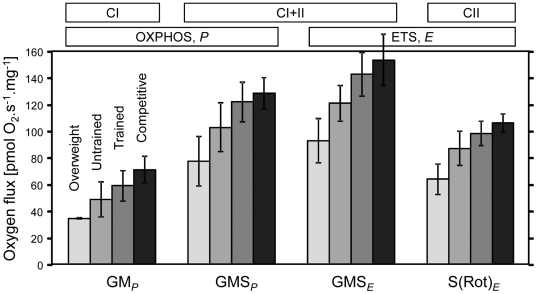
OXPHOS and ETS capacity (pmol·s^−1^·mg^−1^ wet weight) with CI-, CI+II- and CII-substrate supply as a function of physical fitness level (SUIT1 protocol). From light gray to dark gray: overweight-untrained (*N* = 3) → untrained with optimal body condition score (BCS; *N* = 8) → trained with optimal BCS (*N* = 16) → competitive with optimal BCS (*N* = 5); *N*: number of horses.

Maximum tissue mass-specific OXPHOS and ETS capacities with physiological CI+II substrate combinations increased 1.7-fold as a function of BCS and training level (Figure 2). The overweight, untrained and trained groups reached 0.60, 0.80, and 0.94 of the OXPHOS or ETS capacity of the competitive horses.

**Figure 3:**
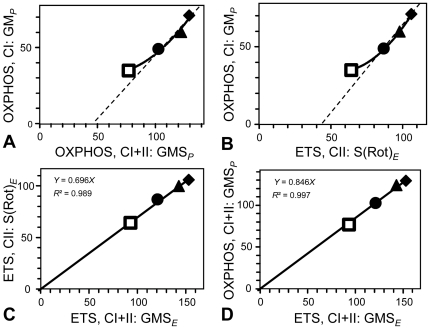
Substrate and coupling control of tissue mass-specific mitochondrial respiratory capacity (pmol·s^−1^·mg^−1^ wet weight) in skeletal equine muscle at different levels of fitness. **A**: Substrate control of CI- *versus* CI+II-linked OXPHOS capacity; CI: glutamate+malate (GM); CI+II: glutamate+malate+succinate (GMS). **B**: Substrate control of CI- *versus* CII-linked respiratory capacity; CII: succinate+rotenone, S(Rot). Dashed lines: linear trend for normal BCS horses, extrapolated to zero; CI-linked capacity increases overproportionally with fitness level. **C**: Substrate control of CII- *versus* CI+II-linked ETS capacity (GMS) increasing in constant proportion with fitness level. **D**: Coupling control of OXPHOS *versus* ETS capacity with CI+II-linked electron input (GMS). The slopes (full lines, forced through zero) provide an estimate of the CII/CI+II substrate control ratio (**C**) and *P/E* coupling control ratio (**D**). All axes are scaled at 110% of the maximum value (average of competitive horses). Open squares: overweight-untrained; closed circles, triangles and diamonds: normal BCS – untrained, trained and competitive, respectively.

CI-linked respiratory capacity increased over-proportionally as a function of physical fitness level, thus indicating a qualitative change of respiratory capacity in addition to the density-dependent effect on OXPHOS and ETS capacity (Figures 3A and B; Table 2). The specific training effect on mitochondrial quality becomes particularly apparent when considering only horses with normal BCS, by excluding the overweight-untrained group: the slopes between CI-linked OXPHOS capacity and either CI+II- or CII-linked respiratory capacities are then apparently linear and non-proportional (significant negative intercept; stippled lines in Figures 3A and B), demonstrating that CI-linked OXPHOS capacity declines more steeply in untrained versus trained horses than CII-respiration. The CI*_P_*/CI+II*_P_* substrate control ratio was significantly higher in competitive horses than in the other groups (Table 3). In contrast, CII-linked ETS capacity increased linearly and proportionally with CI+II-linked ETS capacity as a function of physical fitness, indicating a conserved functional response to changes in BCS and training (Figure 3C). Similarly, the CI+II-linked OXPHOS capacity increased proportionally with CI+II-linked ETS capacity as a function of fitness, such that the *P/E* ratio remained invariable at 0.85 (*N* = 32), indicating a constant limitation of OXPHOS by the phosphorylation system independent of BCS and fitness level (Figure 3D). CI+II-linked and CII-linked respiratory capacities, therefore, qualify as functional markers of mitochondrial density, whereas CI-linked OXPHOS capacity highlights specifically the fact that qualitative changes are superimposed on a shift in mitochondrial density. Taken together, not only tissue-specific capacity but also mitochondrial quality was modified in as a function of physical fitness, as revealed by the substrate control ratios.

**Table 2:** Respiratory parameters from substrate-uncoupler-inhibitor titration protocol 1 (SUIT1) applied on equine permeabilized fibres of *triceps brachii*: OXPHOS and ETS capacities according to fitness level (*N* = 32).

Subgroup	OXPHOS capacity (pmol O_2_.s^−1^.mg^−1^)		ETS capacity (pmol O_2_.s^−1^.mg^−1^)	
	CI: GM*_P_*	CI+II: GMS*_P_*	CI+II: GMS*_E_*	CII: S(Rot)*_E_*
Overweight	35±0	77±18	93±17	64±11
Untrained	49^a^±13	103^a^±18	121^a^±13	87^a^±13
Trained	59^a^±11	122^b^±15	143^b^±16	98^b^±9
Competitive	71^b^±10	129^b^±12	153^b^±19	106^b^±7
**Trained matched SUIT 2&3**	**59±12**	**119±12**	142*±18	**98±9**

*Comments*: between subgroups, respiratory parameters with different letter in superscripts are significantly different (*P*<0.05). No statistical analysis was performed for the overweight group (*N* = 3). Within SUIT1, OXPHOS and ETS capacities with CI+II substrate combinations were significantly different with *P*<0.001 (*). Values are means±SD. For abbreviations, see text.

**Table 3:** Respiratory parameters from substrate-uncoupler-inhibitor titration protocol 1 (SUIT1) applied on equine permeabilized fibres of *triceps brachii*: flux control ratio (*FCR*, with normalization for CI+II*_E_*) and substrate control ratio (*SCR*, assuming CII*_E_* = CII*_P_*); (*N* = 32).

Subgroup	*FCR*			*SCR*			
	CI*_L_*/CI+II*_E_*	CI*_P_*/CI+II*_E_*	CI+II*_P_*/CI+II*_E_* (*P/E*)	CII*_E_*/CI+II*_E_*	CI*_P_*/CI+II*_P_*	CII*_E_*/CI+II*_P_*	CI*_P_*/CII*_E_*
Overweight	0.05±0.01	0.38±0.08	0.85±0.24	0.70±0.12	0.46±0.11	0.84±0.11	0.55±0.09
Untrained	0.04^a^±0.04	0.40^a,b^±0.07	0.85^a^±0.10	0.72^a^±0.09	0.48^a^±0.09	0.85^a^±0.10	0.58^a^±0.17
Trained	0.05^a^±0.03	0.41^b^±0.05	0.86^a^±0.07	0.69^a^±0.07	0.48^a^±0.06	0.81^a^±0.06	0.60^a^±0.09
Competitive	0.04^a^±0.00	0.46^a^±0.03	0.84^a^±0.09	0.69^a^±0.05	0.56^b^±0.08	0.83^a^±0.04	0.67^a^±0.07
**Trained matched SUIT 2&3**	**0.04±0.03**	**0.41±0.05**	**0.84±0.08**	**0.70±0.08**	**0.50±0.08**	**0.83±0.05**	**0.60±0.09**

*Comments*: between subgroups, respiratory parameters with different letter in superscripts are significantly different (*P*<0.05). No statistical analysis was performed for the overweight group (*N* = 3). Values are means±SD. For abbreviations, see text.

### Additive effect of multiple CI-linked substrates and evaluation of SUIT protocols

Addition of 5 mM pyruvate to glutamate+malate resulted in a transient inhibition of OXPHOS, whereas stepwise addition of pyruvate to the final 5 mM concentration was without inhibitory effect (*data not shown*). These observations prompted us to apply a second protocol (SUIT2) in a matched group of trained horses, starting with pyruvate+malate (Figure 1B). The OXPHOS capacity with pyruvate+malate was 1.4-fold higher compared to glutamate+malate. Addition of glutamate exerted a further 1.08-fold stimulatory substrate control effect, which was not masked in the presence of succinate, since OXPHOS capacity was 1.13-fold higher with PMGS compared to GMS. The conventional glutamate+malate and pyruvate+malate substrate combinations, therefore, yield only 0.46 (*N* = 32; *data not reported in the table*) and 0.64 (Tables 4 and 5; trained matched SUIT1 and 2; *N* = 15) of physiological OXPHOS capacity with the PMGS substrate cocktail.

**Table 4:** Respiratory parameters from substrate-uncoupler-inhibitor titration protocol 2 (SUIT2) applied on equine permeabilized fibres of *triceps brachii*: OXPHOS and ETS capacities in trained horses (*N* = 15; trained horses matched with SUIT1 and SUIT3 protocols).

OXPHOS capacity (pmol O_2_.s^−1^.mg^−1^)			ETS capacity (pmol O_2_.s^−1^.mg^−1^)	
CI: PM*_P_*	CI: PMG*_P_*	CI+II: PMGS*_P_*	CI+II: PMGS*_E_*	CII: S(Rot)*_E_*
**83±23**	**90±25**	**134±37**	187*†±39	**96±26**

*Comments*: within SUIT2, OXPHOS and ETS capacities with CI+II substrate combinations are significantly different with *P*<0.001 (*). Between SUIT1 and 2, ETS capacity with CI+II substrate combinations is significantly different with *P*<0.01 (†). Values are means±SD. For abbreviations, see text.

**Table 5:** Respiratory parameters from substrate-uncoupler-inhibitor titration protocol 2 (SUIT2) applied on equine permeabilized fibres of *triceps brachii*: flux control ratio (*FCR*, with normalization for CI+II*_E_*) and substrate control ratio (*SCR*, assuming CII*_E_* = CII*_P_*); (*N* = 15; trained horses matched with SUIT1 and SUIT3 protocols).

*FCR*					*SCR*				
CI:PM*_L_*/CI+II*_E_*	CI:PM*_P_*/CI+II*_E_*	CI:PMG*_P_*/CI+II*_E_*	CI+II*_P_*/CI+II*_E_* (*P/E*)	CII*_E_*/CI+II*_E_*	CI:PM*_P_*/CI+II*_P_*	CI:PMG*_P_*/CI+II*_P_*	CII*_E_*/CI+II*_P_*	CI:PM*_P_*/CII*_E_*	CI:PMG*_P_*/CII*_E_*
**0.04±0.01**	**0.45±0.06**	**0.48±0.06**	**0.71±0.09**	**0.52±0.08**	**0.64±0.07**	**0.68±0.06**	**0.73±0.09**	**0.87±0.16**	**0.94±0.13**

Values are means±SD. For abbreviations, see text.

Tables 6 and 7 report OXPHOS and ETS capacities and coupling control based on CII-substrate only (SUIT3 protocol) and matched with SUIT1 and 2 protocols. The CII-linked ETS capacity was identical in all three protocols (98±9, 96±26, and 97±21 pmol O_2_·s^−1^·mg^−1^ *W*_w_ in SUIT1, 2 and 3, respectively) with intra-individual variability due to tissue heterogeneity [47].

**Table 6:** Respiratory parameters from substrate-uncoupler-inhibitor titration protocol 3 (SUIT3) applied on equine permeabilized fibres of *triceps brachii*: OXPHOS and ETS capacities in trained horses (*N* = 15; trained horses matched with SUIT1 and SUIT2 protocols).

OXPHOS capacity (pmol O_2_.s^−1^.mg^−1^)	ETS capacity (pmol O_2_.s^−1^.mg^−1^)
CII: S(Rot)*_P_*	CII: S(Rot)*_E_*
**95±21**	**97±21**

Values are means±SD. For abbreviations, see text.

**Table 7:** Respiratory parameters from substrate-uncoupler-inhibitor titration protocol 3 (SUIT3) applied on equine permeabilized fibres of *triceps brachii*: coupling control ratio (*CCR*); (*N* = 15; trained horses matched with SUIT1 and SUIT2 protocols).

*CCR*	
*L/E*	*P/E*
**0.32±0.09**	**0.97±0.05**

Values are means±SD. For abbreviations, see text.

### Coupling control and limitation of OXPHOS capacity by the phosphorylation system

Coupling control was independent of fitness, with a mean respiratory control ratio (RCR = *P/L* with glutamate+malate) of 13.5±8.3, and a *FCR* for GM*_L_*/GMS*_E_* of 0.04±0.03 (*N* = 32). The RCR for glutamate+malate and pyruvate+malate were similar (*i.e*. 15.6 and 12.7 in trained matched SUIT1 and 2, respectively; *N* = 15). In contrast, the RCR for succinate+rotenone was only 3.35, which is surprisingly low considering theoretical P:O ratios [48] and the higher capacity of CII- compared to CI-linked respiration.

OXPHOS and ETS capacity (CI+II) increased linearly and proportionally with fitness (Figure 3D). The corresponding phosphorylation system control ratios, *P/E*, were 0.84±0.08 in SUIT1 and 0.71±0.09 in SUIT2 for trained horses (paired data; *N* = 15). With succinate+rotenone, the *P/E* ratio was 0.97±0.05 (Table 7), demonstrating that uncoupling does not significantly stimulate CII-linked OXPHOS capacity.

### Preservation of permeabilized muscle fibres

**Figure 4:**
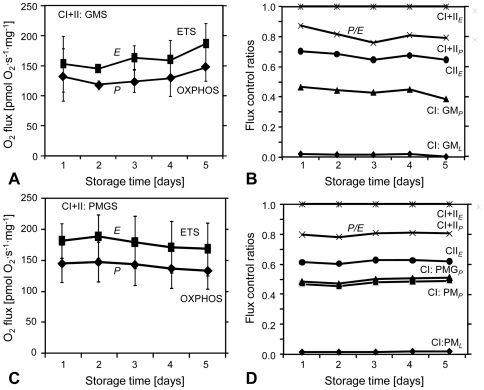
Preservation of respiratory capacities and flux control ratios during extended cold storage of equine skeletal muscle. OXPHOS and ETS capacity (pmol·s^−1^·mg^−1^ wet weight) with CI+II electron input, and corresponding flux control ratios, with protocol SUIT1 (**A** and **B**) and SUIT2 (**C** and **D**). Non-significant changes over a period of 5 days. Each symbol is the mean value ±SD (*n* = 2) for the *N* = 3 horses sampled. All flux control ratios were normalized for CI+II*_E_*.

OXPHOS and ETS capacities with CI−, CII−, and CI+II-linked substrates did not significantly change for 5 days of cold storage of tissue samples nor was there any significant change in *FCR* and *SCR* (Figure 4). Integrity of the outer mitochondrial membrane was well preserved in most of the muscle samples kept in BIOPS at 4°C for up to 5 days, as indicated by the cytochrome *c* test where stimulation of respiration (≤15%) was not significantly increased. From the 60 runs performed in the preservation experiment, nine had to be rejected due to significant cytochrome *c* stimulated respiration (2 at days 1 and 2; 1 at day 3; 1 at day 4; and 3 at day 5). Other signs of mitochondrial alterations observed at day 5 (although without cytochrome *c* effect) were generally reduced respiration except at the level of S(Rot)*_E_*. Low rates of respiration were observed for some samples weighing <1 mg *W*_w_. Only measurements showing none of the above-mentioned signs of mitochondrial alteration were considered in the final analysis.

## Discussion

### Methodological issues

This study applied to horses the methodology used to investigate mitochondrial function in muscle microbiopsy by HRR [27]. Two reference protocols (SUIT1 and 2) were established for horse skeletal muscle. The evaluation and extension of standardized methods is a prerequisite for gaining a new perspective on integrative and comparative mitochondrial physiology.

#### Reference muscle

The *triceps brachii* muscle was selected because of its propulsive and postural role. In horses, the *triceps brachii* is the most important extensor of the elbow and a part of the postural apparatus used to keep the elbow fixed. These roles determine a mixed composition of type I (slow-twitch) and type II (fast-twitch) fibres [32], comparable to human *vastus lateralis* [49], [50]. Importantly, the *triceps brachii* is safer to access for the investigator than the propulsive muscles of the hindlimb. In addition, the hindlimb muscles include a greater proportion (up to 90% in thoroughbred horses) of type II fibres [32], thus rendering them less useful for screening the mitochondrial function.

#### Stability of muscle sample

The equine muscle samples can be stored at 4°C in BIOPS for at least 4 days with good preservation of mitochondrial respiratory function. This storage time is much longer than for human skeletal muscle that can be conserved for only approximately 24 hours [51] or human cardiac muscle with cold storage times limited to 9–12 hours [52]. The stability over several days provides the possibility for transport of muscle samples (under controlled temperature) before analysis of mitochondrial respiration by HRR. Our study involved healthy horses and preservation of mitochondrial function in diseased horses might differ. In case, we recommend to analyze mitochondrial respiration as soon as possible following sampling.

#### Specific experimental conditions and mitochondrial pathways

Respirometric experiments were performed at physiological temperature (37°C), with high ADP concentration, no substrate limitation and with a strict control of the level of oxygen in the oxygraph chambers.

In SUIT1 and 2, the maximum oxygen fluxes obtained after addition of optimal uncoupler concentration indicated that the phosphorylation system exerts control over coupled respiration. On the other hand, the phosphorylation system did not limit CII-respiration since addition of FCCP did not significantly stimulate S(Rot)*_P_*. Therefore, S(Rot)*_P_* may be estimated from S(Rot)*_E_* obtained in SUIT1 and 2.

The low *P/E* ratio in SUIT2 and SUIT1 with CI+II electron supply, and the absence of an uncoupling effect in SUIT3 (*P/E* close to 1) are explained by the low ADP:O ratio with CII as compared to CI electron input, and by upstream limitation of flux when succinate is the sole substrate. The capacity of the phosphorylation system becomes saturated and thus limiting when oxygen flux is higher and more ADP is phosphorylated per oxygen consumed with combined CI+II electron input, involving proton translocation at CI, CIII and CIV. At lower oxygen flux with succinate only, and lower ADP:O with coupling restricted to CIII and CIV, the capacity of the phosphorylation system is sufficiently high to match the ETS capacity, with the consequence of an increase of *P/E* to 1. Similarly, the higher ETS capacity with pyruvate+malate+glutamate+succinate *versus* glutamate+malate+succinate explains the lower *P/E* ratio with PMGS *versus* GMS. SUIT1 and 2 protocols differ by the addition of pyruvate, the product of glycolysis thus studying mitochondrial respiration with (SUIT 2) or without (SUIT 1) assessment of aerobic glycolysis through the activity of the PDH complex.

The minimally invasive character of the sampling technique, the feasibility of the microbiopsy under field conditions, and the long preservation of muscle samples open new perspectives for equine sports medicine, such as the study of the effect of racing by sampling horses before and after a race [26]. The protocols described in this study are proposed as reference guidelines for studies of mitochondrial respiratory control in the equine field, providing the basis for extending investigations to include fatty acid and α-glycerolphosphate oxidation.

### Integrative and comparative physiology

*What is the upper limit of tissue-OXPHOS capacity in triceps brachii muscle of healthy horses and how does it scale with body size and outstanding aerobic capacity of horses?*

Maximal OXPHOS capacity has been rarely measured in trained horses [26]. We found that OXPHOS capacity with CI+II ranged from 60 to 150 pmol O_2_·s^−1^·mg^−1^ *W*_w_, (*i.e.* from 80 to 201 ml O_2_·min^−1^·kg^−1^ *W*_w_) increasing with fitness level from overweight to competitive horses. This is comparable to the range of OXPHOS capacity of human *vastus lateralis* increasing from 60 to 180 pmol O_2_·s^−1^·mg^−1^ *W*_w_ as a function of sedentary-overweight to athletic life styles [8].

With isolated mitochondria and NADH-linked substrates only (including pyruvate), maximal respiratory capacity is equivalent to 115 pmol O_2_·s^−1^·mg^−1^ *W*_w_ (155 ml O_2_·min^−1^·kg^−1^; 37°C) of tissue [25] in accordance with our results on trained horses (Table 2). The highest OXPHOS capacity was obtained in competitive horses (presumably with high 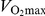) with a calculated mitochondrial oxygen consumption of 4.2 ml O_2_.min^−1^.ml^−1^ mitochondria (assuming a mean mitochondrial volume density based on Kayar *et al*. [23] of 75.10^−6^ ml.mg^−1^). These values are in the range of 3–5 ml O_2_.min^−1^.ml^−1^ mitochondria obtained from morphometric data correlated to 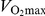 in various mammals [53].

Assuming that 100% of the ergometric 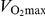 is consumed by skeletal muscle mitochondria in quadrupedal mammals, Kayar and collaborators [23] calculated a mitochondrial oxygen consumption of 4.5 ml O_2_.min^−1^.ml^−1^ mitochondria in three horses at 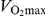 of 60.8 l O_2_.min^−1^. By plotting 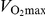 against total muscle mitochondria in several athletic and sedentary species, a mean mitochondrial oxygen consumption of 4.9±0.4 ml O_2_.min^−1^.ml^−1^ mitochondria is found [3], [54]. Therefore, despite the large difference in 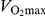 between athletic *versus* non-athletic mammals, mitochondrial oxygen consumption appears to be fixed among species including horses [23], [54].

Our results suggest that higher oxidative capacity in trained *versus* competitive horses is not only obtained by biogenesis of more mitochondria of the same kind. In addition, fine-tuning of mitochondrial quality was observed. CI-linked respiratory capacity was increased in competitive horses to a larger extent than CII-linked respiration, and CI/CI+II but not CII/CI+II substrate control ratios were significantly increased (Tables 2 and 3). Therefore, the additive effect of simultaneous electron flow through CI and CII was not constant. This result extends our observations obtained in a previous study showing an over-proportional increase (+67%; significant with *P*<0.05) of CI-linked respiratory capacity in untrained horses following 10 weeks of endurance training *versus* CII-linked respiration (+24% ; non significant) [26]. Taken together, training in horses stimulates mitochondrial biogenesis resulting in increased mitochondrial densities [55]–[57] within and across species, in the transition from an inactive to physically active life style or evolutionary trait [58]. But contrary to an exclusively quantitative effect on mitochondrial density [58], training exerts an additional influence on mitochondrial quality, as shown by the over-proportional increase in CI-linked respiratory capacity with physical fitness level.

Studies on gene expression in equine skeletal muscle following exercise and training have highlighted the roles of genes responsible for OXPHOS, glucose metabolism and fatty acid utilization [59]–[61], but experimental analyses related to the organization of the ETS in equine mitochondria are still lacking. Hypothetically, OXPHOS capacity might be enhanced by biogenesis and/or a differential regulation of CI *versus* CII since there are evidences that Complexes I and III in mammals associate as supercomplexes that behave like a single unit enhancing electron flow by direct channeling of ubiquinone (for a review, see [62]). Supercomplex organization might also play a role in limiting ROS formation in the trained horse. Also, the possible role of mitochondrial dynamics on energy production starts to be evoked [63] and may contribute to explain our observation.

#### Is maximum OXPHOS capacity influenced by training status and does it contribute to performance?

Histochemical and immunohistochemical assays demonstrate that training improves oxidative capacity in horse muscles [64]–[66]. Results of HRR reflected the fitness level of horses: the lowest OXPHOS capacities were observed in overweight and untrained horses, whereas competitive horses had the highest respirometric parameters.

The 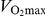 is considered as the gold standard for prediction of athletic ability [67], but performance of horses is also correlated with specific muscle characteristics [68]–[70]. The highest fluxes found in competitive horses suggest a possible link between respirometric parameters and athletic abilities of horses [26]. A greater extraction of oxygen within the working muscle reduces glycolysis and glycogen utilization. Better oxygen kinetics with higher rates of aerobic ATP resynthesis during an effort are decisive to delay fatigue and to ensure performance. Further studies are required to evaluate HRR for performance prediction in specific equestrian disciplines.

#### To which extend are mitochondrial respiratory control patterns in horse skeletal muscle comparable to skeletal muscle mitochondria of mouse, rat and man?

Respiratory capacities obtained with glutamate+malate, pyruvate+malate or pyruvate+malate+glutamate yielded only 50±8%, 64±7% and 68±6% of the flux obtained with further addition of succinate (*N* = 15, trained horses). The additive effect of convergent electron flow through CI+II in equine mitochondria is comparable to skeletal muscle mitochondria of mouse, rat and man, confirming that measurement of the upper limit of tissue-OXPHOS capacity requires reconstitution of the TCA cycle with electron transfer through CI and CII simultaneously [8], [10]–[12].

The *FCR* in SUIT1 and 2 indicated that the phosphorylation system exerts control over coupled respiration. This is similar in human and rat skeletal muscle [10], [11], [71]–[73], but different in mouse skeletal muscle (*P/E* = 1) [13]. In human subjects, a 10-weeks program of endurance and strength training induced an increase of the *P/E* ratio from 0.85 to 0.96 [12]. By comparison, equine OXPHOS capacity was limited by the phosphorylation system to 0.85±0.10 (*i.e.* SUIT1) of ETS capacity, but was independent of fitness level in the present and previous studies [26]. There was a wide range of *P/E* ratios from 0.69 to 0.96 among individuals independent of training status. The magnitude of this apparent excess ETS over OXPHOS capacity might be an important parameter to determine possible capacity for athletic improvement: are trained horses with a *P/E* close to 1 at their maximal capacity of functional adaptation to training? This important question for trainers deserves an answer by conducting additional studies in competitive horses.

Comparative mitochondrial physiology reveals similarities and differences between human and horse skeletal muscle, based on results of this study (and unpublished complementary data; Votion and collaborators) for horses and humans [12]. In both species, CII-linked ETS capacity is an invariant proportion of ETS capacity with convergent CI+II electron input, and the corresponding CII/CI+II substrate control ratio does not change with training. Tissue mass-specific CI+II ETS capacity, therefore, is a functional marker of mitochondrial density, which increases with physical fitness in both species. It is well established that mitochondrial biogenesis is stimulated by training [55]–[57]. However, the study from Pesta and collaborators [12] showed that mitochondrial tissue density is not the sole determinant of increased oxidative capacity per muscle mass. CI*_P_*/CII*_E_* was low in inactive horses (∼0.60) and increased in competitive horses (0.67), where it was still low compared to humans (>0.80). This suggests that there is an apparent excess capacity of CII- over CI-linked respiration in horses, which declined with physical fitness. During a training season of endurance horses, OXPHOS capacity supported by NADH-liked substrates was significantly increased after 10 weeks of endurance training whereas the increase of OXPHOS capacity at the level of CII was less, reaching significance only after an additional period of 10 weeks of training [26]. In human muscle, with a higher CI/CII ratio, CII-linked respiration is more upregulated than CI-linked capacity and this change is already present after 10 weeks of training [12].

In conclusion, horses present adaptations at the level of their muscle mitochondrial function which preclude transposition of results from human studies (*e.g*. training strategies to improve skeletal muscle OXPHOS capacity and aerobic power).

### Perspectives

Results of this study emphasize the potential limiting role of mitochondria in muscle energetics. A prominent characteristic of human patients with OXPHOS disorders is a greatly reduced 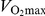 with concomitant deficit in peripheral oxygen extraction [74]. Application of HRR in horses suffering from exercise intolerance provides a powerful diagnostic tool for functional evaluation of mitochondrial disorders.

Recently, gene expression analysis in muscle biopsies of French draft horses suffering from a glycogenosis described as polysaccharide storage myopathy revealed down-regulation of several mitochondrial and nuclear genes involved in OXPHOS [75]. Altered OXPHOS might also play a central role in the pathogenesis of atypical myopathy [76]–[78], a highly fatal environmental condition that affects grazing horses [79]. Our fundamental understanding of the pathophysiology of inherited and acquired equine myopathies will be improved by detailed diagnosis of mitochondrial function, as shown by the direct link between mitochondrial function and physical fitness, including obesity in untrained horses. Ultimately, advances in understanding the role of mitochondrial respiratory capacity in equine fitness and myopathies will extend the conceptual basis required to interpret the diversity of mitochondrial function in other species, including mouse and man.

### Conclusions

This paper proposes a reference method for screening mitochondrial respiratory function in horses. The small amount of muscle obtained by the minimally invasive microbiopsy technique is sufficient for testing the mitochondrial function in Pfi with multiple SUIT protocols using HRR. Standardized methods are described to collect, store and prepare the muscle sample, to measure the mitochondrial function related to different metabolic pathways, and to analyze and interpret the data. This approach appears suitable for the study of OXPHOS capacity in healthy horses and evaluation of training programs in sport horses. In addition, the technique could be used as a first line of functional mitochondrial diagnosis in all suspected cases of equine neuromuscular disorders.
