# Correction: Physical Fitness and Mitochondrial Respiratory Capacity in Horse Skeletal Muscle

**DOI:** 10.1371/annotation/e24ced68-9c2d-4303-9553-0661decb9a51

**Published:** 2012-08-09

**Authors:** Dominique-Marie Votion, Erich Gnaiger, Hélène Lemieux, Ange Mouithys-Mickalad, Didier Serteyn

There is information missing from the Funding section. The complete, correct funding statement is: This study was supported by the 'Ministère de l'Agriculture et de la Ruralité de la Région Wallonne' of Belgium (subvention 06/472 10). H. Lemieux was supported by postdoctoral fellowships from the National Sciences and Engineering Research Council (Canada) and Fonds Québécois de la Recherche sur la Nature et les Technologies (Quebec, Canada). The K-Regio project MitoCom Tyrol administered by E. Gnaiger and OROBOROS INSTRUMENTS received contributions from the Tyrolian Government and the European Regional Development Fund. The funders had no role in study design, data collection and analysis, decision to publish, or preparation of the manuscript. 

